# Living Photoanodes for Solar-Driven Water Oxidation

**DOI:** 10.1021/acs.chemrev.5c00921

**Published:** 2026-02-23

**Authors:** Rachel M. Egan, Angelo J. Victoria, Jenny Z. Zhang

**Affiliations:** † Yusuf Hamied Department of Chemistry, 2152 University of Cambridge, Lensfield Road, Cambridge CB2 1EW, United Kingdom

## Abstract

Photosynthetic microorganisms are abundant, self-sustaining catalysts that convert solar energy into high energy electrons. By interfacing these living catalysts with electrodes, these electrons can be harnessed for electricity generation or chemical production in sustainable solar-powered technologies. The development of these so-called living photoanodes is an emerging and highly interdisciplinary field that has progressed substantially in recent years. In this review, we chart these advancements, beginning with our current understanding of the fundamental biology underpinning the key photocatalytic and electron transport processes of oxygenic photosynthetic microorganisms—namely cyanobacteria. We then describe theoretical approaches to estimating the maximum obtainable photocurrent outputs of living photoanodes to gauge their technological potential. Next, we discuss the main strategies employed to attain these values which include genetic engineering, electrode and diffusional/polymeric mediator design. Finally, in the outlook section, we recommend standardized reporting methods to formalize the field and propose future research directions to realize the full potential of this nascent technology.

## Graphical abstract

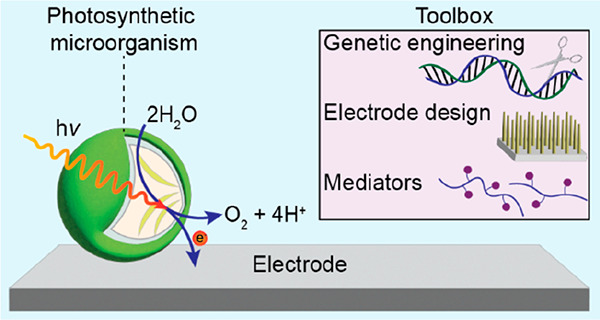

## 1 Introduction

To replace fossil fuels with a sustainable alternative, we must innovate new technologies to rapidly produce energy dense fuels and chemicals using simple and abundant building blocks (H_2_O, CO_2_, N_2_, O_2_). These processes should be entirely powered by renewable energy sources such as solar energy, which delivers enough energy to Earth in just 1 h to meet the annual global energy demand.^1,2^ To be truly sustainable, these technologies must be made of Earth-abundant materials^3^ and can either be readily recycled or readily replenished to avoid the depletion of finite resources.

The natural process of oxygenic photosynthesis—first evolved in ancestral cyanobacteria more than 2.4 billion years ago^4^—satisfies many criteria for this new technology. Using H_2_O as an electron donor, cyanobacteria, algae and plants convert solar energy into a fuel in the form of biomass. Such organisms are abundant, require only H_2_O and CO_2_ as feedstocks, utilize sunlight as their energy source and are self-sustaining. Anoxygenic counterparts, including purple bacteria, green sulfur bacteria, heliobacteria, and filamentous anoxygenic phototrophic bacteria, use alternative electron donors such as organic compounds, sulfur compounds or metal ions. Although there is growing interest in these microorganisms, water is a more ideal substrate for global-scale energy applications.^5^ The distinct bioenergetics and biotechnological potential of anoxygenic microorganisms have been extensively reviewed elsewhere and will not be considered in this review.^6–8^

Despite the advantages of oxygenic photosynthetic organisms in energy conversion, the overall solar-biomass conversion efficiencies are extremely low (<1% for crop plants and <3% for microalgae cultivated in bioreactors) as these organisms have evolved to survive rather than produce fuels for powering modern human activities.^9^ Vast areas of land are required to generate sufficient amounts of energy from biomass, posing a significant threat to agriculture in a world where food security is already a critical issue.^10^ While the natural process is not directly suitable as a sustainable energy solution without re-engineering, it offers a blueprint from which inspiration can be drawn for the creation of new solar-fuel technologies more fit for purpose. This led to the emergence of the field known as artificial photosynthesis in the 1970s which aims to mimic the natural pathway using tailored synthetic materials.^11^ Artificial photosynthetic systems can absorb a wider range of the solar spectrum than natural photosynthetic pigments, especially when complementary synthetic photosensitizers are used in tandem.^9^ They can also exhibit greater solar-electricity or solar-chemical conversion efficiencies due to their relative simplicity and modularity which enable facile control of charge separation and transport.^12–16^ However, artificial systems often rely on expensive or limited resources and exhibit poorer long-term stability, as activity cannot be restored after degradation, limiting scale-up prospects.^17^ Natural systems on the other hand are inherently scalable because of their abundance and capacity for self-repair and reproduction. Artificial systems also lag behind their natural counterparts in terms of their ability to selectively generate a diverse range of complex multicarbon products.^17^ In nature, this ability is conferred by multiple enzymes working in concert as part of a metabolic pathway, all of which have evolved high substrate and product specificity.^18,19^ This level of specificity and product complexity is presently unmatched by synthetic catalysts, which often engage in unwanted side reactions and are generally limited to the production of simpler molecules.^20,21^ Thus, although gains in solar spectrum absorption and solar-electricity conversion efficiencies can be made using purely synthetic systems, they are not yet competitive with their biological counterparts in terms of scalability, selectivity or product complexity.

An alternative solution sits at the interface of biological and artificial photosynthesis: the field of semi-artificial photosynthesis aims to combine the best of biology with state-of-the-art synthetic materials, paving new, more efficient routes for solar-chemical conversion. Typically, in these biohybrid systems, biological catalytic machinery, such as enzymes, membranes or whole cells, are interfaced with either semiconductor nanoparticles in a photocatalytic setup, or electrodes in a photoelectrochemical configuration (Figure 1).^17,22^ By artificially rewiring biological systems in this manner, inefficient steps are supplanted or completely new pathways are forged, achieving solar-chemical conversion efficiencies that surpass natural photosynthesis. System performance can be optimized by incorporating synthetic photosensitizers to improve solar spectrum utilization, and mediators, in the form of diffusional redox species or polymers to enhance charge transfer at the biotic-abiotic interface. The division of labor produces a synergistic effect by assigning tasks (light absorption, catalysis, electron transfer) to the most effective candidate, improving performance by overcoming the limitations of either purely biological or purely artificial systems.

**Figure 1:**
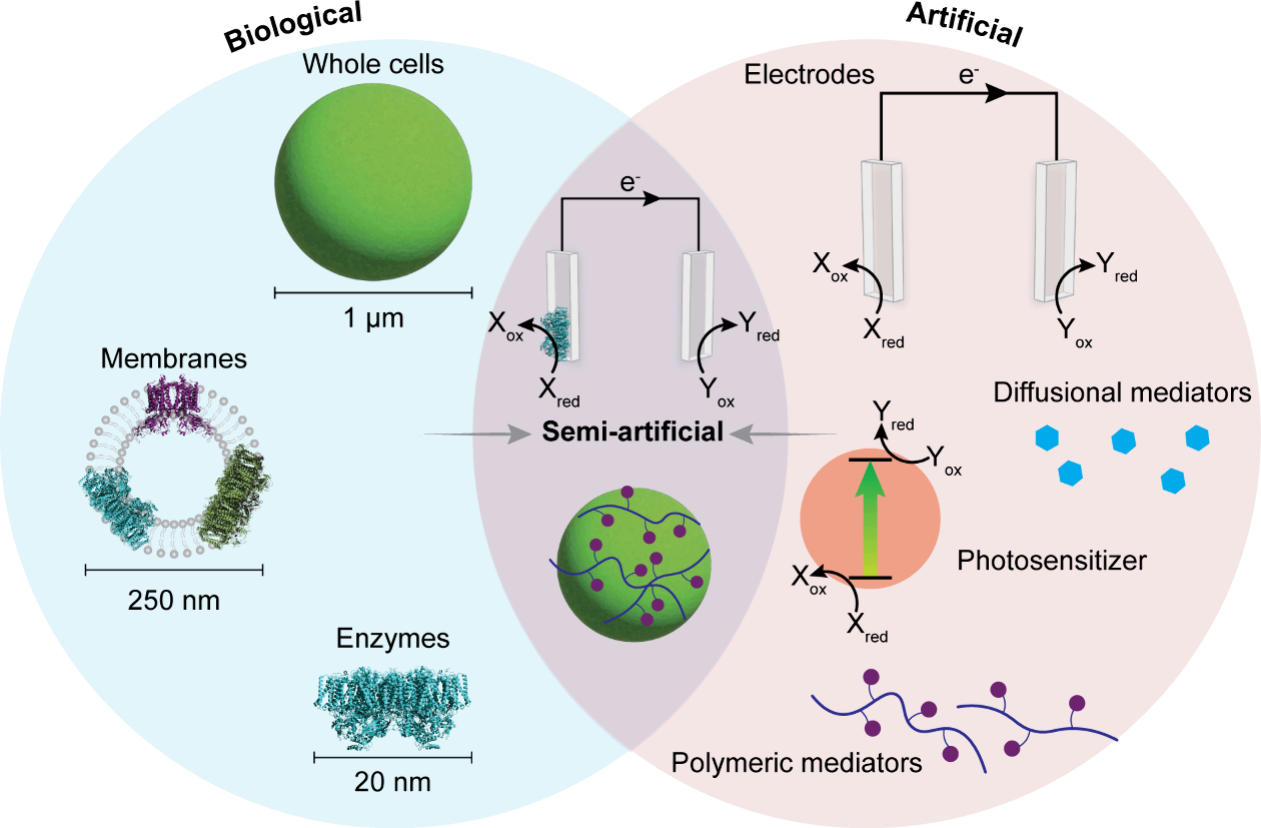
The concept of semi-artificial photosynthesis. Biological and artificial components are combined to produce a synergistic effect. Enzymes, membranes, or whole cells serve as catalysts; electrodes provide modularity and enable new-to-nature reaction pathways; photosensitizers can extend solar spectrum utilization; and diffusional or polymeric mediators enhance charge transfer across the biotic–abiotic interface. The enzyme structures depicted were obtained from the Research Collaboratory for Structural Bioinformatics Protein Data Bank (RCSB PDB): Photosystem II dimer (blue, PDB: 3WU2 ^23,24^ ), Cytochrome *b*_6_ *f* dimer (purple, PDB: 4H44 ^25,26^ ), and Photosystem I trimer (green, PDB: 1JB0 ^27,28^ ).

Although numerous combinations of biological and artificial components are possible, this review specifically focuses on the development of *in vivo*, or living photoanodes for solar-driven water oxidation. These refer to systems that interface whole oxygenic photosynthetic microorganisms (mainly cyanobacteria) as light-driven water oxidation catalysts with electrodes for solar energy conversion applications. Section 2 begins with a comparison of these living photoanodes to simpler enzyme-based systems, where the isolated light-driven water oxidation enzyme, Photosystem II (PSII), is interfaced with electrodes. The section discusses the state-of-the-art PSII-based photoanodes and examines the distinct advantages and challenges of using isolated enzymes vs native membranes vs whole cells as photocatalysts on electrodes. Section 3 provides a comprehensive overview of the fundamental biology underlying the photosynthetic and extracellular electron transfer (EET) pathways in cyanobacteria. It provides up-to-date knowledge of these pathways, how they intersect and key questions that remain unanswered about how photosynthetic electrons are exported outside of the cell. Section 4 discusses current efforts to calculate reliable theoretical predictions of the maximum obtainable levels of photocurrent, which are necessary for setting realistic performance targets, and compares them with existing benchmarks. Section 5 describes the main strategies used to enhance photocurrent outputs toward these targets. These include genetic engineering, intelligent electrode design and the addition of mediators (diffusional or polymeric). The review concludes with the authors’ outlook for the field in Section 6, outlining current bottlenecks and proposing an actionable roadmap to reach targets and facilitate the incorporation of these electrodes into functional real-world devices.

## 2 Photoanodes: From Enzymes to Membranes to Whole Cells

Water is an ideal electron donor for use in sustainable solar energy conversion technologies due to its abundance, nontoxicity, low reducing power and low cost.^5,29^ Oxygenic photosynthesis begins with light-driven water oxidation at Photosystem II (PSII), nature’s only water oxidation enzyme. PSII efficiently absorbs visible light, generating a charge-separated state with a quantum efficiency of >85%.^30^ The hole generated by this photoexcitation, the strongest known biological oxidizing agent, is refilled by electrons liberated by the kinetically and thermodynamically demanding water oxidation reaction (2H_2_O → O_2_ + 4H^+^), catalyzed by the oxygen-evolving complex (OEC) of PSII.^5,31,32^

The catalytic center of the OEC is comprised of a Mn_4_Ca cluster which facilitates the four-electron oxidation of water. The process follows the Kok cycle, in which the OEC progresses cyclically through five metastable states (S_0_, S_1_, S_2_, S_3_, and S_4_), releasing one electron during each step except the final transition (S_4_ – S_0_), where molecular oxygen is evolved.^33,34^ Designing biomimetic water oxidation catalysts is challenging as several aspects of the biological mechanism remain unresolved despite recent progress.^35–37^ These include how water molecules are incorporated, how the O–O bond is formed, and what the protonation states of the OEC and its ligand environment are during catalysis.^38^

Despite this, synthetic water oxidation catalysts now routinely exhibit higher activity than PSII, as evaluated by turnover frequency (TOF), which measures the number of substrate molecules converted per catalyst site per second (Table 1).^39^ However, the native enzyme generally outperforms synthetic catalysts in terms of overpotential, selectivity and stability (Table 1). This is because the performance of PSII has been honed by evolution: the active site (composed of Earth-abundant elements) and surrounding protein scaffold are tuned to stabilize transition states or intermediates through electrostatic/hydrogen bonding, minimizing energy requirements while steric effects control selectivity.^18,19,40,41^ This enables PSII to operate near the thermodynamic limit under mild conditions. *In vivo*, the OEC is reassembled as frequently as twice per hour to replace photodamaged machinery and enable continuous stable operation.^42^ By contrast, many synthetic catalysts require large overpotentials or forcing conditions to drive the reaction, utilize scarce elements such as Ru or Ir limiting their scalability, and cannot be revived once deactivated.

**Table 1:** Comparison of PSII to Synthetic Water Oxidation Catalysts^a^

Catalyst	Abundance	Conditions	TOF (s^–1^)	TON	η (mV)	FE	Ref.
PSII *in vivo* (Mn-based)	abundant	physiological aqueous solution (thylakoid lumen) 5 < pH < 6.5	∼100–400	∼10^6^ (per individual protein, estimated from protein turnover rates; protein turnover *in vivo* enables continuous operation)	<300	100%	^42–46^
Ru-based	rare	mixed CF_3_CH_2_OH/pH 1.0 (v:v = 1:2)	303 ± 9.6	8,360 ± 91	n.r.	n.a.^b^	^47^
Ru-based	rare	phosphate-buffered aqueous solution (0.1 M), pH = 7.2	16,000	4.2 × 10^7^	530	93%	^48^
Ir-based	rare	KNO_3_ (0.1 M), pH = 2.6	7.9	10^6^	520	99%	^49^
Mn-based	abundant	acetate buffer solution (0.1 M), pH = 6	22	13.2	74	93%	^50^
Mn-based	abundant	NaHCO_3_/Na_2_SiF_6_ buffer (50 mM), pH = 5.2	2.84 × 10^–3^	5.2	530	n.a.^b^	^51^
Fe-based	abundant	acetonitrile/water (10:1) mixed solution with Et_4_NClO_4_ (0.1 M), pH = 4.8	1900	10^6^–10^7^	889	96%	^52^
Co-based	abundant	aqueous sodium phosphate (0.2 M), pH = 7	1400	n.r.	570	85–90%	^53^
Cu-based	abundant	phosphate-buffered aqueous solution (0.2 M), pH = 12	267	4.74 ± 0.1	620	97 ± 2%	^54^

a The performance of PSII and a range of representative molecular water oxidation catalysts with different metal centers is assessed in terms of turnover frequency (TOF), turnover number (TON), overpotential (η), and faradaic efficiency (FE). n.a. = not applicable, n.r. = not reported.

b A chemical oxidant was used to drive the reaction so FE is not applicable.

Taken together, PSII combines high activity, selectivity, stability and sustainability, making it an attractive choice for the catalytic component of semi-artificial photoelectrochemical systems. PSII can be interfaced with electrodes as an isolated enzyme, retained within its native membrane structure, or *in vivo* within a living microorganism (Figure 2). In all cases, PSII serves as the catalyst; however, each system has distinct advantages and challenges due to the different local environments.

**Figure 2:**
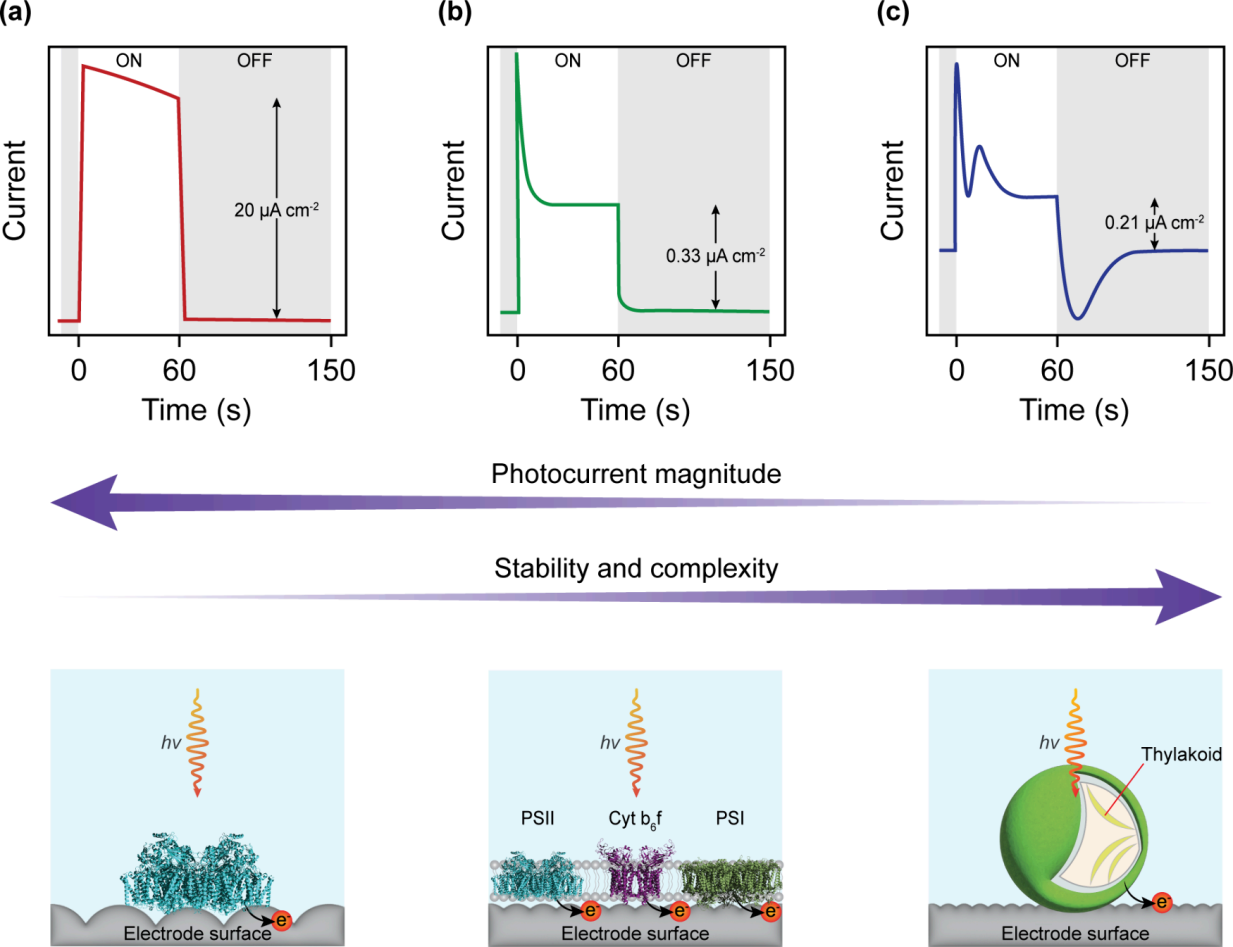
Enzymatic vs membranous vs microbial photoanodes. The typical current profiles of (a) isolated PSII, (b) thylakoid membranes and (c) cyanobacteria interfaced with electrodes under illumination are shown in the top row. The photoelectrochemical output increases in stability and complexity but decreases in magnitude with increasing biological complexity, from isolated enzymes to native membranes to living cells. For isolated PSII and thylakoid membranes, orientation on the electrode is important to ensure the redox active sites interface directly with the surface. The photocurrent densities indicated were taken from Mersch et al.,^56^ Lawrence et al.,^64^ and Zhang et al.^59^ for (a), (b), and (c), respectively. The photocurrent density is defined as the difference between the steady state current in the light and in the dark normalized to the geometric area of the electrode. In all cases, IO-ITO electrodes, red light (λ = 680 nm) and no exogenous mediators were used. The enzyme structures depicted were obtained from the RCSB PDB: Photosystem II dimer (blue, PDB: 3WU2 ^23,24^ ), Cytochrome *b*_6_ *f* dimer (purple, PDB: 4H44 ^25,26^ ), and Photosystem I trimer (green, PDB: 1JB0 ^27,28^ ).

In a PSII-based photoanode, the redox-active sites of the enzyme are readily accessible to the electrode, either directly (if the enzyme is oriented appropriately) or through the addition of mediators which act as electron shuttles. The relatively small size of the enzyme (dimensions of 20 × 10 × 11 nm^55^) also enables high catalyst loading densities to be achieved, particularly if combined with high electroactive surface area electrodes. PSII-based photoanodes have been developed that produce large photocurrent densities up to 930 μA cm^–2^ in the presence of a diffusional mediator.^56^ These photoanodes have also been coupled to hydrogenase^56,57^ and formate dehydrogenase^58^ cathodes in photoelectrochemical systems. By directly coupling water oxidation to hydrogen or formate production using electrodes, thereby establishing artificial pathways, solar-fuel conversion efficiencies surpassing those of natural photosynthesis have been achieved.^56^ However, the integration of enzymes into biohybrids is plagued by stability challenges. Many enzymes are deactivated *ex vivo* over short time scales, especially in aerobic environments,^59^ due to the generation of reactive oxygen species (ROS) which cause irreversible damage to protein structures. This is unavoidable in the case of PSII, which generates oxygen as a product, causing PSII-based photoanodes to destabilize within minutes of operation.^60^ This in turn hampers scalability and real-world implementation. This issue is exacerbated by the extensive enzyme extraction and purification process which is less conducive to scale-up.^17,22,61–63^

An intermediate level of complexity between enzyme-based and living systems arises when PSII embedded in its native thylakoid membrane is interfaced with electrodes (Figure 2(b)). In this configuration, other components of the photosynthetic electron transport chain (PETC) embedded in the membrane can communicate electronically with the electrode, including Photosystem I (PSI), a photosensitizer that re-energizes electrons derived from PSII during photosynthesis. These membrane-based photoanodes demonstrate improved longevity relative to PSII-based photoanodes due to the stabilization of proteins within their native lipid environment. The isolation procedure is also more straightforward and achieving the optimal orientation on the electrode is simplified as the extracted membranes retain their native topology (with the cytoplasmic side facing out).^64^ Unlike the monophasic current profile of PSII-based photoanodes, thylakoid membranes display a sharp spike which decays to a steady state, followed by a rapid return to the steady state dark current once the light is turned off. These distinct features of the profile were attributed to PSI- and PSII-dependent electron transfer processes, respectively.^64^ Most notably, thylakoid-based photoanodes exhibit a very negative onset potential for photocurrent production (1 V more negative than PSII-based photoanodes) enabling high energy electrons to be extracted. These membrane-based photoanodes are valuable tools for probing the fundamental bioenergetics and interplay of the photosynthetic and respiratory electron transport chains which are colocalized in thylakoid membranes in cyanobacteria.^64^ However, the thylakoid extraction process removes key cellular machinery necessary for repairing damage, so long-term operation is yet to be achieved.

These stability issues can be circumvented by using PSII *in vivo* – that is, using oxygenic photosynthetic microorganisms such as cyanobacteria and microalgae as living catalysts. This approach benefits from the inherent self-repair mechanisms of microorganisms to replace damaged cellular machinery, conferring stability and longevity to the system. Cells also self-replicate and can adapt to fluctuating environmental conditions—making them highly robust sustainable/renewable catalysts. The integration of whole cells into biohybrid systems requires coupling their internal metabolism with electrodes. This is a more challenging problem for whole cells compared to isolated enzymes because the catalyst is encapsulated within many layers of insulating cell membranes and extracellular polymeric substances (EPS). Conveniently, many microorganisms including cyanobacteria and microalgae exhibit a phenomenon known as extracellular electron transfer (EET) whereby electrons derived from internal metabolic pathways are hypothesized to be exported to the external environment. When an electrode is present, a biological current can be measured both in the dark and under light irradiation.^65^ Compared to eukaryotic microalgae, prokaryotic cyanobacteria have fewer membranes which act as barriers to EET, making them more conducive to forming effective interfaces with electrodes, generally yielding higher photocurrent outputs (see Table 2).^66^ As a result, they are the predominant catalyst choice for the development of living photoanodes and the focus of this review. However, the large size, dynamicity, heterogeneous surface chemistry, and overall greater complexity of whole cells compared to enzymes present many challenges with interfacing these microorganisms with electrodes. This complexity is reflected in the current profile of cyanobacteria interfaced with electrodes, consisting of a series of peaks and troughs which reach a steady state in both the light and dark phases (Figure 2(c)). The biological origin of this shape is not yet fully understood, but may be due to multiple processes. A study in which the outer layers and extracellular appendages of the cells were systematically removed revealed that the outer membrane and periplasmic space contribute to the additional photocurrent profile features observed for *Synechocystis* sp. PCC 6803 (*Synechocystis*) on inverse opal indium tin oxide (IO-ITO) electrodes that are absent for isolated thylakoid membranes under the same conditions. These layers were hypothesized to play a part in “gating” extracellular electron transfer for whole cells.^67^ A more recent study by Lawrence et al. has shown that isolated native thylakoid membranes interfaced on electrodes show distinctive spike features that relate to electron kinetics from the PETC.^64^

**Table 2:** Photocurrent Outputs of Cyanobacteria-, Microalgae-, Thylakoid-, and PSII-Based Photoanodes in the Literature^a^

	Biocatalyst	Electrode	Photocurrent density (μA cm^–2^)	Applied potential (mV vs SHE)	Mediator	Year	Ref.
Whole cell (cyanobacteria)	*Leptolyngbya sp.*	graphite	48.2	550	POs and FeCN	2014	^100^
	*Synechocystis*	IO-ITO	14.7	500	DCBQ	2018	^59^
	*Synechocystis*	graphite	5.1	500	PEDOT	2020	^101^
	*Synechocystis*	micropillar ITO	245	500	DCBQ	2022	^97^
	*Synechocystis* (GM)	carbon paper	30	450	None	2022	^77^
	*Synechocystis*	graphite	2.5	500	PDA and FeCN	2024	^102^
	*Synechococcus elongatus PCC 7942*	flat ITO	0.83	500	PEDOT-CPE and FeCN	2025	^103^
Whole cell (microalgae)	*Paulschulzia pseudovolvox*	graphite	11.5	550	POs and BQ	2015	^104^
	*Chlamydomonas reinhardtii*	carbon gauze	64.0	850	DCBQ	2018	^105^
	*Chlorella vulgaris*	Au	5	550	POs	2022	^106^
	*Chlorella minutissima*	WO_3_	12.0	700	PDA	2025	^107^
Thylakoid membranes	Thylakoid (*Spinacia oleracea*)	AuNP–Au	130	600	para-BQ	2014	^108^
	Thylakoid (*Spinacia oleracea*)	graphite	42.4	500	POs	2015	^109^
	Thylakoid (*Spinacia oleracea*)	AuMP–screen-printed carbon electrodes	62.5	600	POs	2018	^110^
	Thylakoid (*Spinacia oleracea*)	micropatterned carbon on quartz	71	200	[Ru(NH_3_)_6_]^3+^	2018	^111^
	Thylakoid (*Synechocystis*)	IO-ITO	3.3	700	FeCN	2025	^64^
Isolated PSII	PSII (*Thermosynechococcus elongatus*)	IO-ITO	930	500	DCBQ	2015	^56^
	PSII (*Thermosynechococcus elongatus*)	IO-ITO	230	500	POs	2016	^112^
	PSII (*Spinacia oleracea*)	nanotubular ITO film with Au paste	39	500	DCBQ	2017	^113^
	PSII (*Thermosynechococcus elongatus*)	IO-ITO	185	500	DCBQ	2018	^59^
	PSII (*Thermosynechococcus elongatus*)	IO-ITO	80	–200	POs and DPP (photosensitizer)	2018	^57^
	PSII (*Thermosynechococcus elongatus*)	IO-graphene	12.3	500	DCBQ	2019	^114^
	PSII (*Thermosynechococcus elongatus*)	ITO NPs	22.5	550	POs	2020	^115^
	PSII (*Spinacia oleracea*)	macroporous CN	77.4	841	DCBQ	2025	^116^

a Abbreviations: BQ = benzoquinone; CN = carbon nitride; DCBQ = 2,6-dichloro-1,4-benzoquinone; DPP = diketopyrrolopyrrole; FeCN = ferricyanide; GM = genetically modified; IO = inverse opal; ITO = indium tin oxide; MP = microparticle; NP = nanoparticle; PEDOT-CPE = poly­(3,4-ethylenedioxythiophene)-conjugated polyelectrolyte; PDA = polydopamine; POs = osmium-based redox polymer; SHE = standard hydrogen electrode.

## 3 Electron Transport Pathways in Photosynthetic Microorganisms

### 3.1 The Photosynthetic Electron Transport Chain (PETC)

The implementation of living photosynthetic organisms in biohybrid systems requires a deep fundamental understanding of their physiology, particularly in relation to photosynthetic and EET activity. In cyanobacteria, the light reaction of photosynthesis occurs in thylakoid membranes which are located in the cytoplasm and are semibound to the plasma membrane.^68^ A simplified scheme of the photosynthetic electron transport chain (PETC) and its associated bioenergetics are shown in Figures 3 and 4, respectively. The process begins with light absorption by antennae proteins (not shown) which funnel energy to the reaction center, the special chlorophyll *a* pair, P680 (λ_max_ = 680 nm), of PSII. Electrons derived from water oxidation are transferred from the oxygen-evolving complex (OEC) of PSII to refill the hole generated by photoexcitation.^31,32^ The release of protons during this step contributes to a proton gradient established across the thylakoid membrane during electron transfer downstream of PSII which drives the synthesis of adenosine triphosphate (ATP) by ATP synthase.^22,69^ Meanwhile, the excited electron is transferred via pheophytin to plastoquinone A (Q_A_) and finally to plastoquinone B (Q_B_) which dissociates from PSII and diffuses through the thylakoid membrane, entering the plastoquinone (PQ) pool. Electrons are transferred from the PQ pool to the transmembrane protein Cytochrome *b*_6_ *f* (Cyt *b*_6_ *f*) which reduces plastocyanin (Pc) that diffuses through the thylakoid lumen to Photosystem I (PSI).^70^ PSI (a photosensitizer rather than a photocatalyst) contains a reaction center, special chlorophyll *a* pair, P700 (λ_max_ = 700 nm) which upon light absorption and subsequent electron transfer via a series of cofactors, achieves a relatively long-lived charge-separated state with almost 100% quantum efficiency.^32,71,72^ The hole generated by photoexcitation is filled by oxidation of Pc and meanwhile the terminal electron acceptor of PSI, an Fe–S cluster (F_B_) transfers electrons to ferredoxin (Fd) on the cytosolic side.^73^ From here, the pathway diverges depending on the metabolic needs of the cell.^32,71^ For example, electrons may be transferred to ferredoxin-NADP^+^ reductase (FNR) to generate nicotinamide adenine dinucleotide phosphate (NADPH) which feeds electrons into the Calvin-Benson-Bassham (CBB) cycle for carbon fixation.^70,71^ Electrons can also be rerouted during cyclic electron transport where electrons from PSI are fed back into the PQ pool, to control the ATP/NADPH balance and provide photoprotection. To add further complexity, the respiratory electron transport chain (RETC) in cyanobacteria partially overlaps with components of the PETC such as the PQ pool, Cyt *b*_6_ *f* and Pc in the thylakoid membrane.^71^ For clarity, the RETC and other related pathways are omitted from Figure 3; further details can be found in the following references.^64,71^

**Figure 3:**
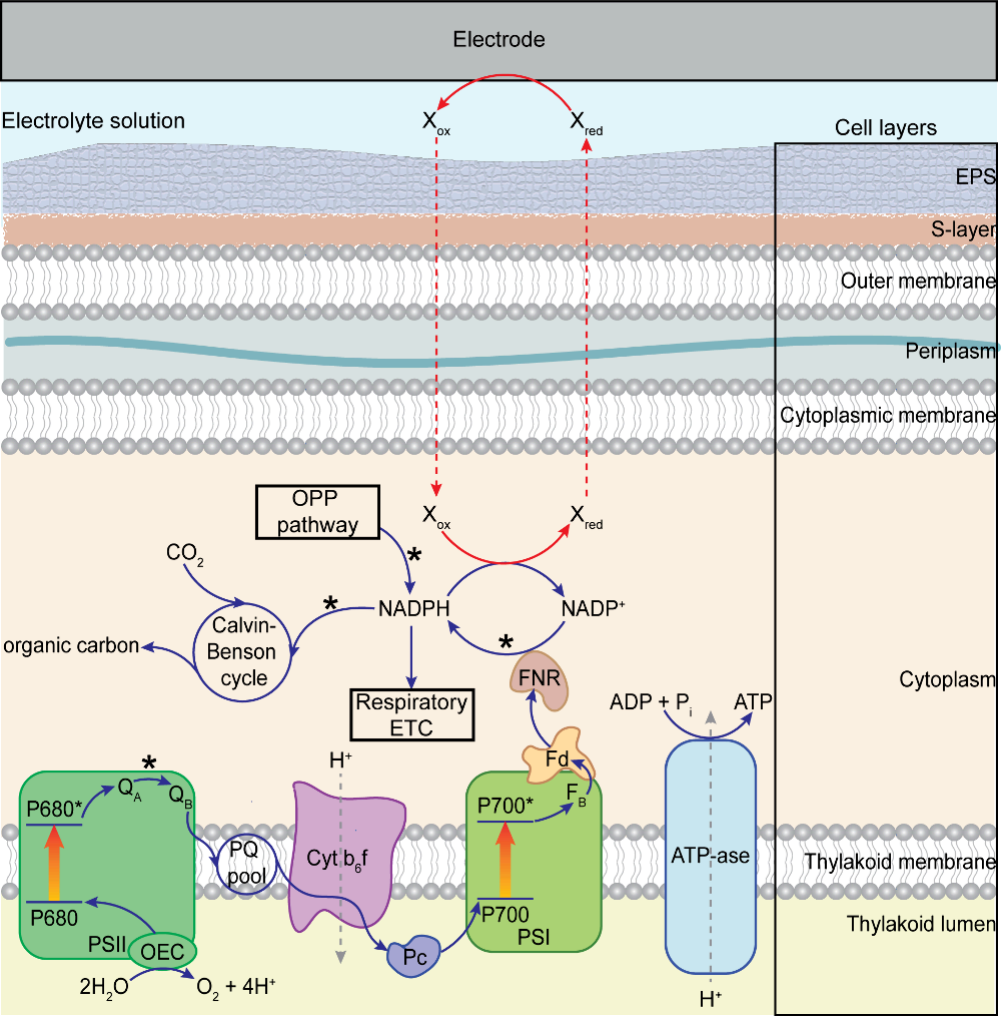
Simplified PETC and EET pathway in cyanobacteria. Electrons liberated from water oxidation in the oxygen-evolving complex (OEC) of Photosystem II (PSII) are transferred along the chain via the plastoquinone (PQ) pool, Cytochrome *b*_6_ *f* (Cyt *b*_6_ *f*), plastocyanin (Pc), Photosystem I (PSI), and ferredoxin (Fd), culminating with NADPH generation by ferredoxin-NADP^+^ reductase (FNR). The proton gradient established across the thylakoid membrane during electron transport is used to drive ATP synthesis by ATP synthase (ATP-ase). NADPH may be used for respiration by the respiratory electron transport chain (RETC), carbon dioxide fixation by the Calvin-Benson-Bassham (CBB cycle) or it may reduce the proposed small diffusional mediator (X_ox_/X_red_) involved in EET. The oxidative pentose phosphate (OPP) pathway is also implicated in EET, through its role in generating NADPH in the dark. The exact mechanism by which X_ox_/X_red_ interacts with the PETC and is transported across the cytoplasmic membrane, the periplasm (which includes a peptidoglycan layer), the outer membrane, the surface layer (S-layer) and the extracellular polymeric substances (EPS) is unknown. Points in the pathway that have been inhibited chemically or through genetic modifications to investigate the EET pathway are marked with an asterisk (*).

**Figure 4:**
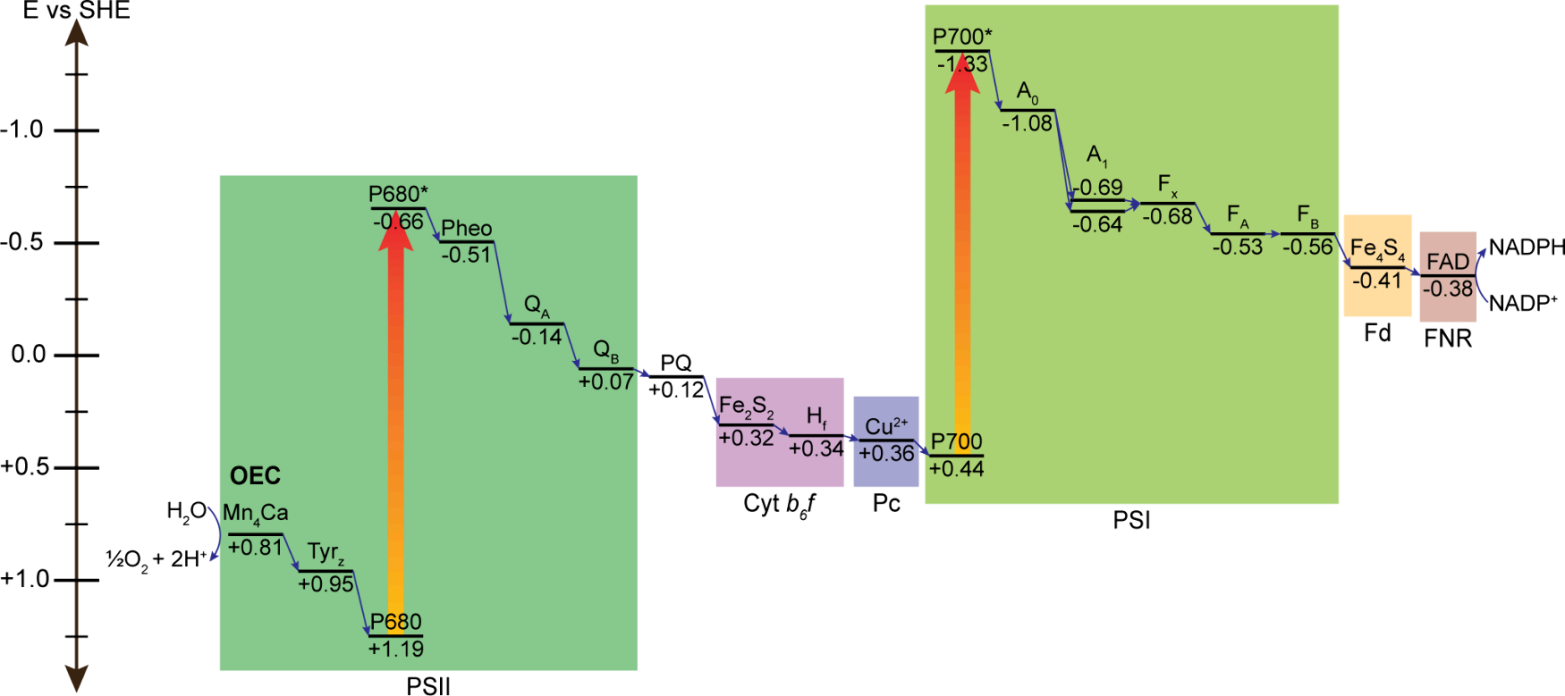
Energetics of the photosynthetic electron transport chain in cyanobacteria (Z-scheme). The approximate midpoint potentials of the various redox-active components are shown.^5^ Abbreviations: A, acceptor; Cyt, cytochrome; F, Fe; FAD, flavin adenine dinucleotide; Fd, ferredoxin; FNR, ferredoxin-NADP^+^ reductase; H, haem; NADPH, nicotinamide adenine dinucleotide phosphate; OEC, oxygen-evolving complex; P, primary electron donor; Pc, plastocyanin; Pheo, pheophytin; PQ, plastoquinone; PSI, Photosystem I; PSII, Photosystem II; Q, quinone; SHE, standard hydrogen electrode; Tyr, tyrosine.

### 3.2 The Extracellular Electron Transfer (EET) Pathway

Cyanobacteria interfaced with electrodes produce complex photocurrent profiles under illumination, indicating the PETC and EET pathways are interlinked (Figure 2(c)).^66,67,74^ The precise mechanism and function of EET in cyanobacteria remains to be elucidated. EET can be categorized as either direct, where electron transfer occurs via conductive cellular appendages such as pili, or indirect, via endogenous diffusional redox mediators secreted by the cells. It is widely accepted that in cyanobacteria, electrons exported during EET are primarily derived from water oxidation by PSII, the EET pathway intercepts the PETC downstream of PSI and that the final electron acceptor is a diffusional redox species.^75^ The putative pathway is shown in Figure 3; however, the full sequence of steps involved is only partially understood.

The use of site-specific inhibitors to block electron transfer from PSII to Q_B_ and PSII-less mutants have shown that PSII is the primary source of electrons that are exported from cyanobacteria during EET.^74,75^ Neither inhibition nor removal of PSII completely abolished the photocurrent. In both cases, the residual photocurrent was attributed to respiratory electrons because of the functional overlap between the photosynthetic and respiratory electron transport chains.

Evidence for indirect EET was obtained by Zhang et al. and Saper et al., who both independently observed reversible redox waves in cyclic voltammograms of cyanobacteria on electrodes under light irradiation, indicative of a small diffusional redox species such as a quinone or flavin.^59,69^ Numerous studies have reported that either permeabilization^69^ or genetic removal of the outer layers of the cells, including the extracellular polymeric substances^76^ or the outer membrane,^77^ boosted photocurrent outputs by up to an order of magnitude, suggesting that these structural layers hinder EET. By disrupting or removing these layers, more endogenous mediators are released by the cells, further bolstering the hypothesis that the mechanism of EET in cyanobacteria is indirect. Saper et al. confirmed the diffusional nature of the mediator by showing its ability to bypass a 3 kDa dialysis membrane, suggesting it is a small, soluble molecule rather than a protein.^69^ Wey et al. showed that the photocurrent produced by cyanobacteria biofilms is diminished by introducing stirring, thereby replacing the diffusional layer with mediator-less electrolyte solution. The photocurrent is reinstated once stirring is stopped, providing complementary evidence of the involvement of an endogenous diffusional mediator in EET.^76^ A direct EET mechanism via pili has been ruled out by knockout mutant studies that demonstrate photocurrent production in cyanobacteria is pili-independent, though they do play a role in cell attachment to electrodes.^67,78^

The point at which the PETC and EET pathways intersect has been determined to be downstream of PSI using chemical inhibitors. Blocking electron transport beyond PSI completely eliminates the photocurrent output suggesting the reducing end of PSI is the exit point of electrons destined for EET.^75^ Kusama et al. more specifically pinpointed NADPH as the electron donor to the endogenous mediator based on their observation that the photocurrent is enhanced in the presence of a CBB cycle inhibitor.^77^ In this scenario, NADPH that would normally enter the CBB cycle is rerouted to the EET pathway. Although NADPH has been proposed as the diffusional redox mediator itself,^79^ other studies have refuted this hypothesis.^77,80,81^ NADPH was further implicated in the EET mechanism by Hatano et al. using mutants of the oxidative pentose phosphate (OPP) pathway, which produces NADPH in the dark. Impeding this pathway reduced the availability of NADPH and significantly diminished photocurrent production. Maintenance of a pool of NADPH by respiratory and photosynthetic electron transport chains and the OPP pathway is essential for EET activity. The identity of the diffusional redox mediator and exact pathway for its export beyond NADPH remains to be elucidated and is an area of active research. Our limited understanding of the pathway makes it challenging to identify effective rewiring strategies for boosting output.

Nonphotosynthetic exoelectrogenic microorganisms utilize EET to respire anaerobically, using extracellular transition metal oxides as terminal electron acceptors.^82^ Although cyanobacteria utilize molecular oxygen as a terminal electron acceptor during aerobic respiration, the potential involvement of transition metals in EET in cyanobacteria should not be overlooked. It has been hypothesized that the alternative respiratory oxidase (ARTO), located in the cytoplasmic membrane, plays a role in EET by reducing iron—a potential electron carrier—in an assimilatory pathway.^83^ However, this has been refuted by a study showing that deletion of ARTO enhances EET in the presence of an iron-based diffusional mediator (ferricyanide).^84^ This suggests that ARTO acts as an electron sink that competes with EET. Two recent studies have implicated manganese as contributing to the current response of living photoanodes. Lai et al. showed that oxidation of manganese present in the cell growth medium generates an abiotic dark current.^85^ Kusama et al. reported that photosynthetically induced increases in local pH at the electrode surface promote manganese oxidation, potentially contributing to the photocurrent output.^77^ As manganese is an essential component of the OEC of PSII, exploring the interplay between EET and manganese homeostasis could provide new insights into the mechanism and physiological role of EET in oxygenic photosynthetic microorganisms.^86^

## 4 Theoretical Photocurrent Outputs

Establishing the theoretical maximum photocurrent outputs of photosynthetic machineries on electrodes is important for assessing the performance of current systems, identifying bottlenecks and devising strategies to overcome them. Lawrence et al. estimated that maximum photocurrent densities of approximately 10 mA cm^–2^ could be achieved by a rewired photosynthetic electron transport chain on an electrode.^5^ To put this value into context, this current density is within an order of magnitude of that produced by a conventional single junction silicon solar cell (≈42 mA cm^–2^) operating close to the theoretical limit (the Shockley Quissler limit).^5,87–89^ In this calculation, the rewired PETC consists of a PSII–PSI complex randomly close-packed on a flat electrode. Diffusional limitations to the overall rate were not considered and the system was subject to standard solar simulation conditions (AM1.5G, tilted 37°).

However, this estimate does not represent a realistic scenario for whole cells. McCormick et al. provided a more conservative estimation which considered intact cyanobacterial cells in a three-dimensional (3D) biofilm within a porous transparent electrode, using sunlight as the sole energy source.^90^ Their calculations predicted achievable current densities ranging from 340 μA cm^–2^ – 2,400 μA cm^–2^ based on the average incident solar energy at locations far north of vs near to the equator (approximately 10% and 26% of AM1.5G, respectively). The two main assumptions underpinning the higher-value calculation are: (1) that only 2–3% of photosynthetically derived electrons are required for essential metabolic activities,^91^ leaving the remainder available for EET; and (2) that these electrons are collected by electrodes with a faradaic efficiency of 60–95%. This latter assumption is based on reported faradaic efficiencies for heterotrophic microorganisms transferring electrons derived from the oxidation of organic substrates to electrodes,^92–95^ which may not be directly translatable to cyanobacteria. While these assumptions are optimistic (only a small fraction (<1%) of photosynthetic electrons are allocated to EET in actuality),^84,96^ they highlight the two key areas that need to be addressed to improve outputs. These include the redirection of intracellular electron flux to the EET pathway, and ensuring electrons that are exported are efficiently collected by the electrode.

Neither estimate accounts for the potential of photocurrent enhancement strategies to improve performance beyond these theoretical maxima. These include protein engineering to increase catalysis rates, photosensitizers to improve solar spectrum utilization or increase voltages, and mediators to improve electron transport rates.

At present, the benchmark photocurrent density for cyanobacteria on electrodes is 245 μA cm^–2^ which falls just outside the theoretical range predicted by McCormick et al. (Table 2).^97^ This corresponds to an external quantum efficiency (EQE), the proportion of photons converted into electrons collected by the electrode, of 29%. Although the current state-of-the-art is 2–3 orders of magnitude below the theoretical maximum, it mirrors the early stage development of dye-sensitized solar cells 30 years ago—whose performance has significantly improved over time following numerous advancements.^5,98^ Photoelectrochemical systems that employ organic semiconductors have also seen an analogous improvement in photocurrent outputs, progressing from the μA range to the mA range since the 1980s through the implementation of various strategies.^99^ Similar progressions in our ability to redirect electron flow toward the EET pathway, increase cell loading densities, improve solar spectrum utilization, limit photoinhibition effects, and increase cell–electrode electron transfer efficiencies will elicit rapid gains in the output of cyanobacteria on electrodes in the near future.

## 5 Strategies to Enhance Photocurrent Outputs

Both biotic and abiotic approaches can be employed to improve the photocurrent output from cyanobacteria electrodes. These include genetic engineering, electrode design and the use of mediators or nanomaterials. Although these strategies are implemented to achieve a common goal, they operate by different mechanisms. Genetic engineering aims to manipulate the biological machinery to enhance EET, primarily through the removal of electron sinks or physical barriers to EET, or through the addition of non-native EET pathways. Electrode design involves the optimization of the electrode structure and surface chemistry to promote high catalyst loading densities, strong cell–electrode interactions and optimal light flux. Diffusional mediators function by extracting additional electrons from the PETC, while polymeric mediators facilitate efficient electron transfer at the cell–electrode interface. While the effectiveness of each of these strategies has been studied extensively, the implementation of multiple strategies in concert in the future could reveal unanticipated synergistic effects. These strategies are discussed in detail in the following sections.

### 5.1 Genetic Engineering

Genetic engineering approaches to enhance photocurrent outputs from photosynthetic microorganisms can be broadly categorized into three strategies. These include: (1) modulation of cellular redox metabolism toward EET, (2) modification of physical barriers to EET, and (3) introducing heterologous EET machinery from other microorganisms. The model organism *Synechocystis* is widely used in biohybrid systems because its full genome has been sequenced and it is genetically tractable, making it particularly amenable to genetic engineering.^117^ Recently, faster-growing strains of cyanobacteria have been identified that are also genetically tractable, facilitating engineering efforts. However, at present, these have primarily been engineered for elevating biofuel synthesis rather than enhancing EET.^118^ Although EET in oxygenic photosynthetic microorganisms remains relatively less understood than in heterotrophic metal-reducing bacteria such as *Geobacter* or *Shewanella*, many genetic targets have been identified and tested with varying degrees of success.

#### 5.1.1 Modulation of Cellular Redox Metabolism toward EET

One of the first reported strategies for enhancing photosynthetic EET through genetic engineering involved redirecting internal reducing equivalents toward the EET pathway by inactivating competing electron sinks. Targeted knockouts of terminal respiratory oxidases, such as cytochrome *c* oxidase (COX), alternative respiratory terminal oxidase (ARTO), and cytochrome bd-quinol oxidase (Cyd), have been shown to increase the availability of electrons for export.^84,119^ Mutants lacking flavodiiron proteins (Flv1/Flv3), which normally dissipate excess electrons through the reduction of oxygen, also showed increased electron export under fluctuating light.^120^ Genetic modifications that influence the cellular redox balance or eliminate competing pathways offer a degree of control over photosynthetic electron partitioning, funneling current toward EET.

#### 5.1.2 Modification of Physical Barriers to EET

A critical barrier to efficient EET in cyanobacteria is the presence of multiple insulating cell layers which encapsulate the photosynthetic machinery. Some bacterial taxa perform EET through conductive pili or nanowires which span the outer membrane, providing a route for electrons to travel from internal metabolic pathways to the external environment. Although *Synechocystis* produces type-IV pili, they do not play a role in EET, with pili-deficient mutants showing no difference in photocurrent output relative to the wild-type.^78^ A recent report on the conditional repression of the outer membrane using CRISPR interference (CRISPRi) in *Synechocystis* led to an order-of-magnitude enhancement in photocurrent output.^77^ CRISPRi is a genetic technique whereby individual genes are selectively repressed by a protein (dCas), which is guided to its target by a single-guide RNA complementary to the gene’s sequence.^121^ This finding suggests that the outer membrane constitutes a major bottleneck for native or engineered EET pathways, and that structural modification to increase its permeability could substantially increase electron export from photosynthetic microorganisms. This is corroborated by a report that the outer membrane of *Synechocystis* exhibits intrinsically low permeability (20-fold lower than *E. coli*).^122^ Furthermore, it has been shown that removal of the outermost cell layer—the extracellular polymeric substances (EPS) layer—results in a 4-fold increase in photocurrent due to improved cell packing on the electrode. It is hypothesized that the endogenous mediator may also be sequestered or impeded by the matrix of EPS as it diffuses to the electrode surface. The diffusion-slowing, sorptive properties of the EPS is well-reported, although this theory requires further validation.^123^ Alternatively, the biosynthesis of EPS is carbon-intensive so its removal could allow for a greater proportion of electrons to be diverted to the EET pathway.^76,124^ Reducing the physical barriers surrounding the cell is a promising strategy to enhance EET, although striking the right balance to maintain cell viability will be crucial.

#### 5.1.3 Introducing Heterologous EET Machinery from Other Microorganisms

EET machinery from highly exoelectrogenic bacteria can be introduced, providing a new route for electron export (Figure 5(a)). The outer membrane cytochrome OmcS involved in EET in *Geobacter sulfurreducens* have been successfully heterologously expressed in *Synechococcus elongatus*.^125,126^ In their native host *Geobacter sulfurreducens*, these cytochromes self-assemble into extensive nanowire structures that transport electrons over several micrometers.^127,128^ While OmcS protein expression has been confirmed in cyanobacteria, formation of fully assembled nanowires has not yet been observed. This may be challenging to achieve, as the biosynthesis of conductive nanowires imposes a substantial metabolic burden. In particular, the high iron requirement of multiheme nanowires may directly compete with iron allocation necessary for the maintenance of photosynthetic machinery in cyanobacteria.^129^ Phenazine biosynthesis machinery from *Pseudomonas aeruginosa* has been expressed in *Synechocystis*, enabling the cells to produce non-native electron mediators.^130^ Although this approach did not lead to photocurrent enhancement, it clearly demonstrates that cells can be engineered to regenerate electron mediators, negating the need for their replenishment in real-world applications.

**Figure 5:**
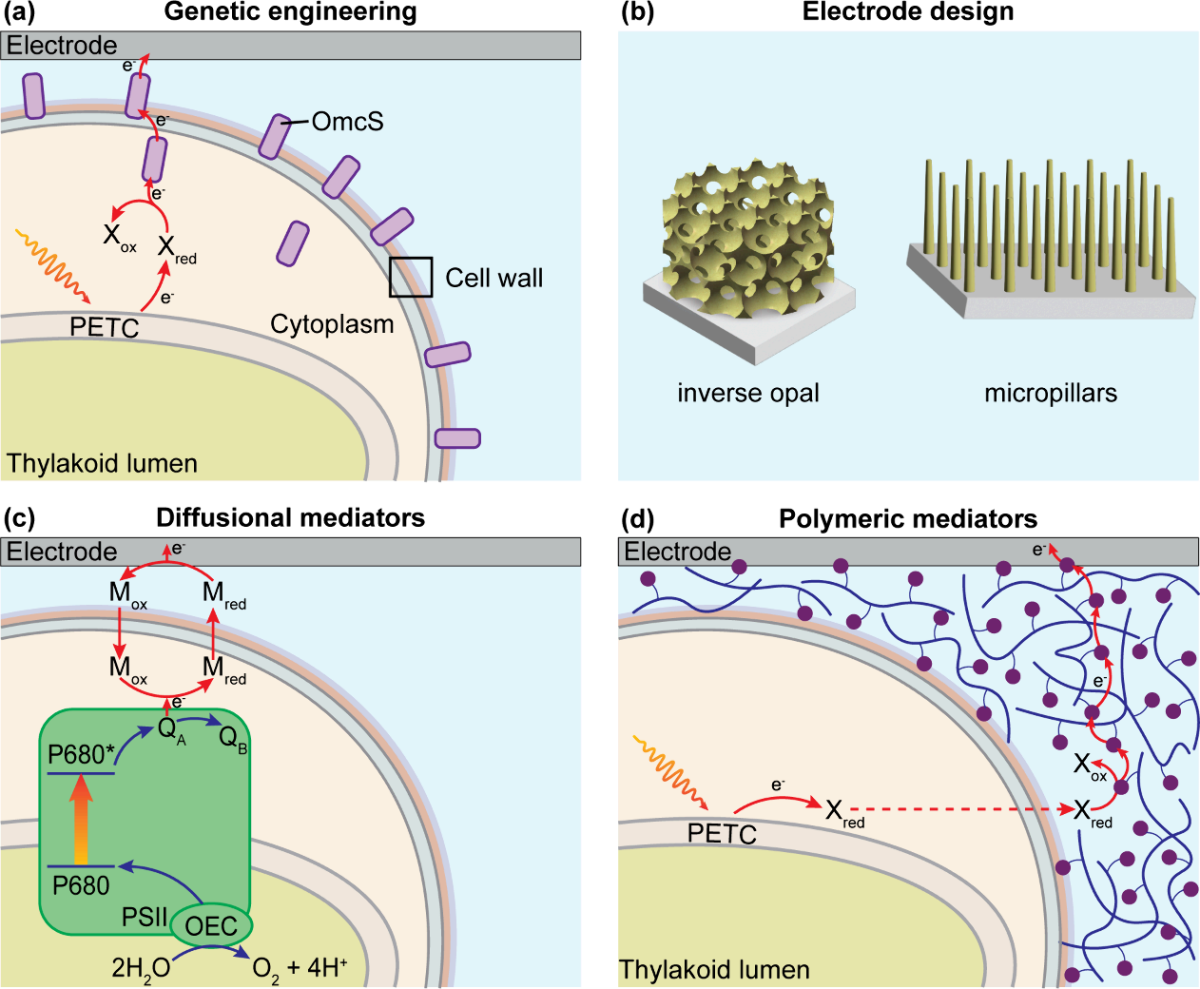
Rewiring strategies for boosting photocurrent outputs from cyanobacteria on electrodes. (a) Genetic modification of cyanobacteria. In this example, the expression of EET machinery, outer membrane cytochrome S (OmcS), from highly exoelectrogenic microorganisms creates a new pathway for electron export. (b) Intelligent electrode design includes state-of-the-art inverse opal indium tin oxide (IO-ITO) and micropillar ITO electrodes which improve cell loading and light management. (c) Diffusional mediators directly extract photosynthetic electrons from the PETC and deliver them to the electrode. (d) Polymeric mediators form a conductive bridge to improve charge transfer efficiency at the cell–electrode interface.

Our incomplete understanding of the key components in the EET pathway presents a significant challenge in identifying the most effective genetic modifications to increase photocurrent outputs without negatively impacting key cell functions. The practical use of genetically modified organisms in applications may be hindered by regulatory hurdles associated with the use of antibiotic resistance markers. To overcome this, strategies for creating markerless mutants, i.e. genetic modifications that do not rely on antibiotic selection, should be employed. These include counterselection systems or CRISPR/Cas-based genome editing.^131,132^

### 5.2 Electrode Design

Due to the prevalence of planktonic systems in the literature, where an electrode is immersed in a suspension of photosynthetic cells, electrode design has often been overlooked as a photocurrent enhancement strategy. However, the most substantial progress toward approaching the theoretical maximum photocurrent output has been achieved through advancements in electrode design for biofilm systems, where cells are directly interfaced with the electrode surface. This configuration of living photoanodes can yield larger photocurrent outputs but is more challenging to characterize due to the complex microenvironments established at the cell–electrode interface. In biofilm systems, both the electrode material and structure impact the performance of living photoanodes primarily through influencing biocatalyst loading and light utilization (factors that are less critical for planktonic systems).^59,66,97^ The ideal electrode material for living photoanodes is highly conductive, inert, biocompatible, transparent or translucent, nontoxic, and resistant to mechanical stress. While metal oxides such as indium tin oxide (ITO) offer many of these desirable properties,^59,97,133^ recent research has increasingly focused on carbon-based electrode materials to promote scalability and sustainability. These include graphite,^100^ carbon cloth,^74^ graphene films,^134^ carbon nanotubes,^135^ pyrolytic carbon,^111^ and carbon nitride.^116^ However, the conductivity and light transmission properties of carbon electrodes must be improved if they are to outcompete their metal oxide counterparts.^136^

The electrode structure should feature a high electroactive surface area (EASA) to accommodate high biocatalyst loading densities and efficient light management properties. First generation electrode geometries were typically flat, leading to poor cell attachment and consequently low outputs.^66^ In the early 2000s, researchers adopted the use of electrodes with nano- or microscale roughness which favored biofilm formation. A major breakthrough was achieved using a templating method to produce 3D hierarchically structured inverse opal indium tin oxide (IO-ITO) electrodes (Figure 6(a)). This design strategy, commonly employed for generating photonic crystals, was later successfully adapted as an electrode structure in the biosensing field, due to the provision of a high internal electroactive surface area.^137–140^ These electrodes feature multilayers of interconnected macropores with nanoscale roughness to aid cell adhesion, leading to high cell loading densities. The synthesis method for IO-ITO is amenable to adaptation and structures with different pore sizes can be fabricated to suit the dimensions of the biocatalyst.^59,66,133^

**Figure 6:**
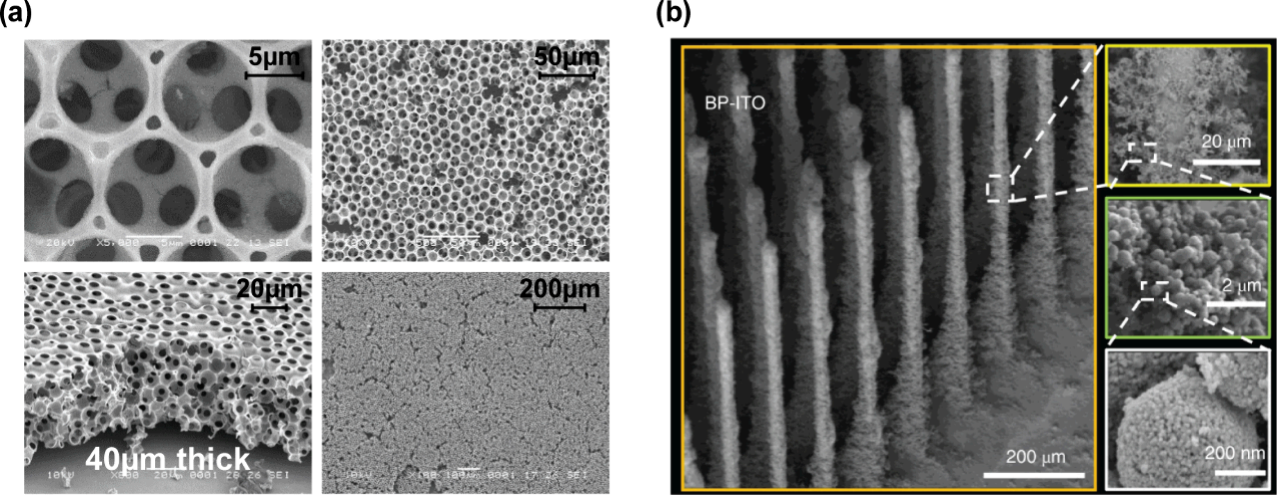
State-the-art electrodes for living photoanodes. (a) IO-ITO electrodes. Reprinted with permission from Zhang et al.^59^ Copyright 2018 American Chemical Society. (b) branched micropillar ITO (BP-ITO) electrodes. Reprinted with permission from Chen et al.^97^ Copyright 2022 Springer Nature.

The current state-of-the-art electrodes for cyanobacteria are branched micropillar ITO electrodes (BP-ITO), fabricated by aerosol jet printing (Figure 6(b)). These electrodes consist of pillar arrays with microscale branches which aid cell loading and boost light-trapping, producing photocurrents of 245 μA cm^–2^ with the assistance of an exogenous diffusional mediator.^97^ By altering various parameters such as pillar height, thickness, spacing and surface roughness, the best-performing micropillar structure was found to exhibit superior light flux, highlighting that this should be a key research focus in electrode design. Notably, these BP-ITO electrodes outperformed IO-ITO structures in spite of the latter exhibiting higher EASA and cell loading. This structure–performance analysis highlights that electrode engineering focused solely on increasing cell loading is insufficient to obtain maximal photocurrent output if cells are poorly connected to the electrode surface or lack adequate access to incident light.

Recent developments in 3D fabrication techniques have positioned carbon-based materials as promising candidates for cyanobacteria electrodes. Innovative designs that enhance light penetration can overcome the challenge associated with the inherent opacity of the material. Structured carbon electrodes with tunable lattice sizes or porous networks have been produced for various applications by integrating methods such as photolithography and additive manufacturing with pyrolysis.^111,141–144^ Micropatterned carbon electrodes generated in this fashion have demonstrated among the highest photocurrent outputs for thylakoid membranes.^111^ A recent study also reported a scalable method for fabricating PSII-based photoanodes using macroporous carbon nitride electrodes, achieving geometric areas of up to 33 cm^2^.^116^

The ideal electrode architecture which maximizes cell loading without compromising light utilization is still being explored. Establishing clear structure–performance relationships will be key to steering future design efforts.^97^ This will be aided by advancements in high throughput methods for generating and testing large libraries of electrodes.

### 5.3 Diffusional Mediators

The addition of exogenous electron mediators boosts photocurrent outputs by “stealing” electrons from the PETC and shuttling them to the electrode surface (Figure 5(c)). This process redirects electrons destined for other metabolic activities toward current generation, benefiting both planktonic and biofilm systems.

An ideal electron mediator for rewiring *in vivo* PETCs should possess several key properties. It should have a low midpoint potential to minimize energy losses^130^ and exhibit fast electron transfer kinetics.^145^ It must be cytocompatible and resistant to photodegradation or chemical modification to ensure stability and long-term functionality.^130,146^ Additionally, the mediator should be membrane-permeable to access intracellular components of the PETC, while also being sufficiently hydrophilic to dissolve in high concentrations in aqueous solutions.^147^ Finding the optimal balance of these sometimes conflicting traits can be challenging.

Commonly employed artificial mediators include inorganic species such as ferricyanide,^75,96^ and organic species such as quinones^59^ and phenazines.^130^ Ferricyanide, which is lipid-insoluble, exhibits good mediation capabilities by penetrating the outer membrane through porins and accepting electrons from within the periplasm.^75,96,148^ In comparison, lipid-soluble quinone mediators such as the state-of-the-art 2,6-dichloro-1,4-benzoquinone (DCBQ) produce much higher photocurrent enhancements (up to 127-fold).^97^ The high performance of DCBQ is partially attributed to its ability to directly extract high energy electrons from the terminus of PSII as well as excited peripheral chlorophyll molecules on the picosecond time scale.^149^ Phenazines, a class of secondary metabolites produced by *Pseudomonas* spp. can also effectively act as mediators of photocurrent from the PETC. Phenazines can extract higher energy electrons due to their lower midpoint potential. However, this also renders reduced phenazines more susceptible to deleterious side reactions, including ROS production, which compete with photocurrent generation.^130^

The activity of high-performing diffusional mediators is typically short-lived for a variety of reasons. Common modes of deactivation include sequestration in cellular compartments,^150^ kinetic quenching,^105^ generation of cytotoxic ROS,^151^ susceptibility to nucleophilic attack and transformation into a lipid-insoluble species which can no longer mediate.^152–154^ In a mechanistic study on the physiological impact of diffusional mediators on *Synechocystis*, Yuan et al. attributed the transient photocurrent enhancement produced by 1,4-benzoquinone and [Co­(bpy)_3_]^2+^ to chemical instability and interrupted electron transfer to PSI, respectively.^155^ These mechanisms severely reduce the lifetime of the system and are poorly understood. Interventions to overcome the trade-off between high photocurrent output and stability should be a major research focus in the future. An in-depth analytical study of DCBQ degradation pathways has revealed the detrimental role of semiquinone radical intermediates formed through both biotic and abiotic processes in solution. This led to the design of a mitigation strategy using “redox helpers” to suppress the degradation of quinone mediators by preventing the build-up of reactive semiquinone radicals.^156^ More general challenges associated with the use of diffusional mediators include environmental contamination, short circuiting issues and the need for replenishment or continuous stirring to maintain activity. These factors increase costs and reduce scalability prospects.^90,157,158^

### 5.4 Polymeric Mediators

Polymeric mediators in bioanodes serve as electron collectors, providing a direct and efficient route for electrons exported from the cells to reach the electrode (Figure 5(d)). By effectively extending the electrode surface, the polymer captures a greater proportion of exported electrons, overcomes the sluggish kinetics of endogenous diffusional species and minimizes losses incurred by their entrapment within the biofilm matrix.^76^ This is particularly beneficial for 3D biofilms on electrodes, where endogenous mediators are released at substantial distances from the electrode surface due to the large size of the biocatalysts.

Polymers can be classified as redox-active, conductive, or a hybrid of both (Figure 7). Redox polymers are comprised of discrete redox species tethered to an insulating polymer backbone through a covalent, coordinate or electrostatic bond. Conducting polymers are comprised of delocalized electronic states arising from a conjugated backbone.^159^ Hybrid redox-conducting polymers refer to structures which combine the two, achieved by either tethering redox-active species to a conductive backbone or by incorporating them directly into the backbone itself.^160,161^ The mechanism of charge transport varies depending on the type of polymer employed. In redox polymers, electron transfer occurs via self-exchange reactions between adjacent redox species in different redox states. This is referred to as Marcus-type, collisional electron transfer or electron hopping.^162^ In conductive polymers, charge transfer occurs via band-type transport, referring to movement of electrons in delocalized molecular orbitals. Electron hopping can also be observed in conductive polymers, especially between chains in disordered systems.^163^

**Figure 7:**
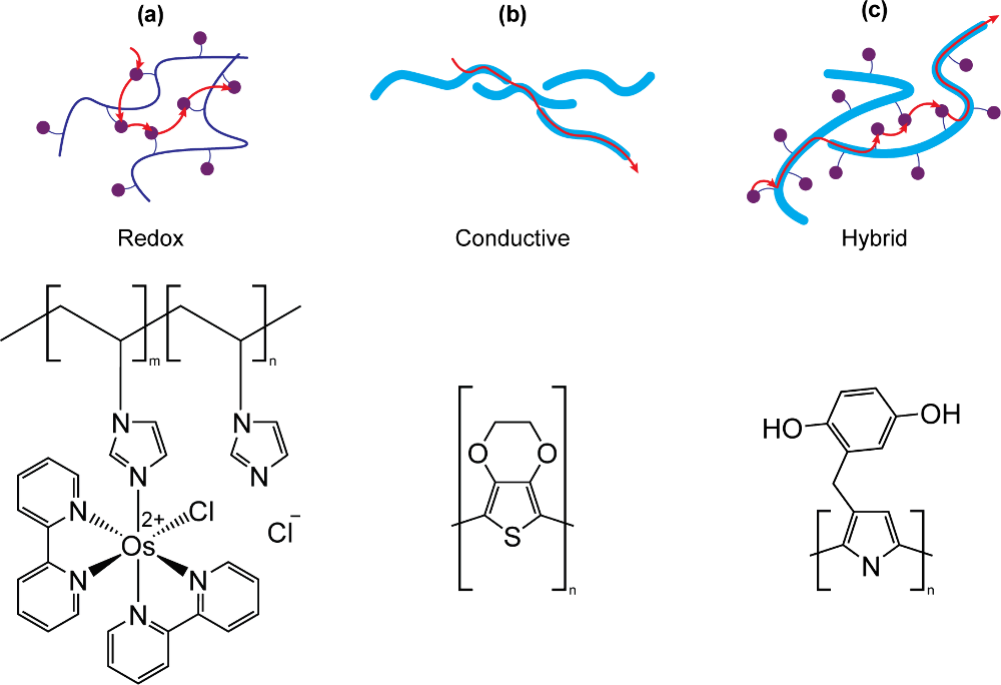
Polymeric mediators. (a) Redox polymers such as an osmium-based redox polymer comprised of an [Os­(2,2’-bipyridine)_2_Cl]Cl complex tethered to a poly­(1-vinylimidazole) backbone. The nonconductive polymer backbone is represented by a thin navy line, with redox-active osmium complexes shown as purple circles. (b) Conductive polymers such as poly­(3,4-ethylenedioxythiophene) (PEDOT). The conductive backbone is represented by a thick blue line. (c) Hybrid conducting redox polymers such as poly­(pyrrol-3-ylmethylhydroquinone). Redox-active hydroquinone species (purple circles) are tethered to a conductive backbone (thick blue line).

Beyond functioning as electron conduits, polymeric mediators in biohybrid systems provide numerous additional benefits. They can enhance biocatalyst loading by serving as an immobilization matrix, entrapping cells on the electrode surface and preventing their detachment over time.^160,164,165^ This is particularly useful in applications such as membrane-less or implantable devices, where leaching of these components must be avoided. Similarly, polymeric mediators are well-suited for prolonged operating times since they do not require replenishment which is advantageous from a sustainability perspective. Polymeric mediators may be less cytotoxic than their diffusional counterparts due to restricted access to photosynthetic machinery, attributed to their larger size preventing them from penetrating cell membranes.^147^ The modular nature of polymers and synthetic flexibility means that they can be precisely tailored through rational design to optimize performance. This includes fine-tuning the midpoint potential, incorporating functional groups for adhesion to electrodes or cell surfaces, and enabling cross-linking to aid stability.^72,160,166,167^

Due to their fast electron transport kinetics, osmium-based redox polymers are considered state-of-the-art and have been extensively implemented in many biohybrid systems to successfully wire a range of biocatalysts to different types of electrodes.^72,100,112,165,168–176^ Osmium-based redox polymers have produced enhanced photocurrent outputs from the cyanobacterium *Leptolyngbya* sp. CYN82 on graphite electrodes (8.64 μA cm^–2^ vs 1.30 μA cm^–2^)^100^ and the green alga *Paulschulzia pseudovolvox* on graphite electrodes (0.44 μA cm^–2^ vs 0.02 μA cm^–2^).^169^ To improve the sustainability of photosynthetic biohybrids, research has extended to more scalable, Earth-abundant redox polymers, based on naphthoquinone.^165^

The organic conductive polymers poly­(3,4-ethylenedioxythiophene) (PEDOT),^101^ polypyrrole,^177^ and polydopamine^102^ have been successfully implemented as polymeric mediators for living photoanodes. Reggente et al. systematically optimized PEDOT:SDS coated graphite electrodes to enhance the photocurrent output from *Synechocystis* by 6-fold.^101^ Liu et al. used PEDOT:PSS to wire *Synechocystis* to a carbon cloth anode in a microscale biosolar cell that demonstrated a consistent high power output over a 20-day period.^178^ Similarly, Chen et al. developed a 3D biocomposite by embedding the photosynthetic cyanobacterium *Synechococcus elongatus* PCC 7942 within a conjugated polyelectrolyte (CPE) matrix derived from PEDOT, resulting in a 10-fold increase in photocurrent production per cell. CPEs are a class of conductive polymers bearing ionic side chains along the backbone, which can be harnessed to construct 3D architectures through interchain ionic interactions.^103^ Polypyrrole coatings have also been implemented as a wiring tool for cyanobacteria, with fibrillar nanostructures proving to be more effective mediators than granular ones.^179^ The efficacy of polypyrrole has also been shown to be specific to the strain of cyanobacteria due to differences in strength of the cell–polymer interaction, highlighting the importance of precisely tailoring interfaces to the microorganism and electrode material in question.^177^ An emerging approach is the *in situ* encapsulation of photosynthetic cells by the self-polymerization of dopamine under mild conditions. This strategy establishes pathways for long-range electron transport and negates the separate synthesis and subsequent integration of the artificial matrix into a biofilm, which is not as conducive to scale-up. Polydopamine has been successfully coated onto *Synechocystis,* ^102^ as well as the purple bacteria *Rhodobacter sphaeroides* ^180^ and *Rhodobacter capsulatus*,^181^ to increase electrode adhesion and charge extraction by 3 to 20-fold. The full encapsulation of photosynthetic cells by a conductive polymer introduces the problem of light penetration which may become a limiting factor, especially in a multilayered 3D electrode architecture.

The incorporation of polymeric mediators into living photoanodes is a promising strategy for increasing photocurrent outputs with significant application potential. While there have been notable successes, the field is still lacking a holistic overview of what makes a polymer an effective wiring tool. This knowledge gap hinders the rational design of optimized polymers, specifically tailored to enhance the photocurrent outputs of cyanobacteria. Direct, systematic comparisons of different polymer types in the same system are scarce and due to the lack of standardization across different studies, it is difficult to gain a mechanistic understanding of how the polymers improve the reported systems. In particular, the elucidation of whether increased photocurrent outputs are due to an enhanced biocatalyst loading effect or a mediation effect is often not well distinguished. The interaction of the polymer with the biocatalyst itself is relatively unexplored, especially in the case where whole cell catalysts are used. Microorganisms exhibit much more complex surface chemistry than isolated photosystems, especially in biofilms, due to the production of EPS. This necessitates targeted studies to understand how polymers interact within these complex local environments. The use of polymers in 3D electrode structures is also rarely studied. In real-world applications, multiple enhancement strategies are likely to be used, necessitating their simultaneous testing to ensure compatibility and effectiveness. To advance living photoanodes beyond proof-of-concept toward real-world applications, longevity studies are needed on the capacity of polymers to facilitate stable photocurrent outputs over long time scales and their impact on cell physiology.

## 6 Outlook

### 6.1 Standardization of Reporting Methods

Although the potential of living photoanodes is clear, significant improvements in performance are needed to reach targets and facilitate their incorporation into devices. To meaningfully assess the performance of living photoanodes, standardization across the field in terms of reporting outputs is required as well as accurate theoretical predictions of maximum performance metrics against which results can be measured.

#### 6.1.1 Light Sources

At present, variability in testing conditions, device set-ups, configurations (planktonic vs biofilm) definitions of performance metrics and reporting methods make it difficult to compare results fairly across studies, necessitating standardization. In particular, the characteristics of the illumination source should be clearly reported, including the lamp model or spectral profile, as these strongly influence performance. Broad-spectrum white light or solar-simulators provide a closer approximation to natural sunlight and are valuable for assessing practical applicability. However, light-emitting diodes (LEDs) with defined wavelengths are preferable for mechanistic studies as they enable the deconvolution of wavelength-dependent effects. The light intensity should ideally be reported in dual units: W m^–2^ which is directly relevant for device benchmarking as well as in μmol_photons_ m^2^ s^–1^, the convention in the photosynthesis field which is more biologically relevant due to the quantum nature of photosynthetic light reactions.^182^ In photovoltaic research, AM1.5G is the internationally standardized solar spectrum conventionally applied at an intensity of ∼1000 W m^–2^, equivalent to ∼6500 μmol_photons_ m^2^ s^–1^.^183^ However, biological photosynthesis can only absorb light in the 400–700 nm range (known as photosynthetically active radiation, PAR) which accounts for a small proportion of the solar spectrum (431 W m^–2^ or 1980 μmol_photons_ m^2^ s^–1^). In addition, AM1.5G does not represent the realistic solar spectral irradiance at the Earth’s surface which is typically only a fraction of this value (1% – 50%) and varies considerably depending on location, season and time of day.^184^ Biological photosynthesis operates more efficiently at moderate or subsaturating light intensities and is hindered by photoinhibition at excessive intensities. Taken together, these considerations indicate that AM1.5G is not a suitable illumination condition for living photoanodes. More realistic and physiologically relevant light intensities for cyanobacteria range from 30–1000 μmol_photons_ m^2^ s^–1^ in the photosynthetically active range.^185^

#### 6.1.2 Defining the Photocurrent Magnitude

Typically, the performance of living photoanodes is assessed by the photocurrent magnitude, which has not been precisely or unanimously defined in the field. Here, we propose to explicitly define the photocurrent magnitude to be the difference between the steady state current under illumination and the steady state current in the dark (Figure 2(c)). This standardization is necessary due to the complex photocurrent profiles produced by photosynthetic microorganisms on electrodes and to avoid misleading readers by reporting transient peak photocurrents which do not reflect the consistent output capacity of the electrode. The photocurrent magnitude is most commonly reported as a photocurrent (A), or more ideally, as a photocurrent density (A m^–2^). Using units that encompass the geometric electrode area enables direct and fair comparison across different studies and provides a more relevant metric for assessing scale-up potential. As the implementation of 3D electrodes becomes more prevalent due to their critical role in maximizing photocurrent output, there are advantages to reporting current normalized to the electroactive surface area in addition to the geometric area (2D areal dimension), when possible. Geometric area is important for defining reaction parameters, such as regions of total photon or electron flux. Normalizing current to geometric area is critical for assessing device-level performance and efficiency. EASA is an important metric that directly influences the catalyst loading capacity of an electrode, and reporting current as a function of EASA distinguishes electrodes that increase outputs primarily through higher catalyst loading from those that enhance performance via other mechanisms such as improved catalytic activities or cell–electrode interactions. The overall system performance of living photoanodes will require a combination of optimal catalyst loading, cell–electrode interaction and highly active biocatalysts. Hence, making this distinction when reporting current densities would be useful for more precisely delineating electrode structure–bioactivity relationships, which would aid holistic system development. A control measurement should also be carried out with the bare electrode to account for abiotic contributions to the photocurrent.

#### 6.1.3 Normalization to Biocatalyst Loading

Normalization of the photocurrent density to the amount of biological material either loaded on the electrode (for a biofilm system) or present in the electrochemical cell (for a planktonic system) should ideally be reported as an extra means of characterization. This can be determined indirectly by measuring the chlorophyll *a* (Chl *a*) content on the electrode, a common proxy for photosynthetic cell number^119,186^ or PSII abundance (adjusted for PSI, based on known ratios under controlled conditions).^187,188^ This method is appropriate when the cell type and physiology is kept constant in the study. Direct reporting of cell number as an additional normalization would be preferable, since Chl *a* levels per cell vary under different conditions,^189,190^ though this is only possible for planktonic systems, and much more challenging to achieve for biofilm configurations.^191^ The Chl *a*-normalized photocurrent density is a good measure of the quality of the interfacial wiring, enabling a distinction to be made between systems where many cells do not contribute to the current and those in which each cell is well-connected. It is important that both metrics are reported as the absolute photocurrent is relevant for device benchmarking, whereas cell- or Chl *a*-normalized values provide insight into the wiring efficiency. The growth phase of the photosynthetic microorganism should be controlled and consistent across experiments to avoid discrepancies due to variations in cell loading.

#### 6.1.4 Additional Parameters

An important parameter frequently omitted from studies is the external quantum efficiency (EQE), which indicates the proportion of incident light converted into current. EQE is an important metric for unveiling light management issues and indicating how efficiently the electrode converts light energy into electrical energy. This value is especially relevant in the later stages of technology development and is necessary for calculating solar-electrical or solar-chemical conversion efficiencies in photoelectrochemical devices.

The stability of the photocurrent output over time is often not reported despite stability being one of the distinct advantages of living electrodes. This is an important parameter informing the feasibility and technological readiness of these systems. As with all biological studies, results should be reported as the mean ± standard deviation of at least three biological replicates to ensure results do not derive from unspecific genetic anomalies.

### 6.2 Modeling Realistic Theoretical Maximum Values

At present there are two theoretical estimates in the literature of the maximum obtainable photocurrent from cyanobacteria on electrodes. These predictions differ greatly in magnitude, with one suggesting an upper limit of 10 mA cm^–2^ and the other suggesting a range of 0.34–2.46 mA cm^–2^ depending on light conditions. To obtain these values, both approaches drastically simplify the system and rely on many assumptions (such as ignoring diffusional limitations or assuming faradaic efficiencies of EET similar to those of heterotrophic microorganisms). Building a more detailed model of the system which fully encapsulates the cyanobacterial metabolic network (using flux balance analysis^192,193^ ), incorporates realistic electrode geometries and accounts for light management would produce more reliable predictions. Such a model would also aid in disentangling the photocurrent profile. However, this is a nontrivial task due to the high level of complexity of the multicomponent system and will require continuous refinement as we accumulate knowledge about the EET mechanism in photosynthetic microorganisms. This knowledge can be used to inform the model, while results from the model can be revelatory and guide experimental design to probe unanswered fundamental questions. Using modeling as a bottom-up approach to predict maximum output can also provide insights into potential bottlenecks in the system, which could be addressed using the aforementioned multidisciplinary strategies.

### 6.3 Future Directions of Photocurrent Enhancement Strategies

While artificial strategies, namely genetic engineering, electrode design and the use of diffusional or polymeric mediators, have been successfully implemented to enhance photocurrent outputs of living photoanodes, further advancements are needed. The gaps that must be filled and potential avenues to address them emerge from assessing the current state-of the-art in the literature, discussed in this review.

Genetic engineering approaches are inherently limited by our incomplete understanding of the identity and function of the endogenous mediator(s), the intersection of EET with other metabolic pathways and its transcriptional and metabolic regulation. Future efforts to uncover this information could take inspiration from the discovery of elusive endogenous diffusional mediators in other microorganisms, such as 2-amino-3-carboxy-1,4-naphthoquinone (ACNQ) in *Shewanella oneidensis MR-1* which was identified using a combination of isolation and analytical methods.^194^ Techniques such as high-performance liquid chromatography (HPLC), mass spectrometry, and Raman spectroscopy could also prove revelatory and have yet to be applied to the search for the structure of the endogenous mediator(s) in photosynthetic microorganisms. Genetic methods that control the expression of individual genes of interest could be a powerful tool to determine their effect on the photocurrent output in a top-down approach.

The main challenge in electrode design for living photoelectrodes is to produce electrodes from sustainable materials without sacrificing conductivity or effective light management. This could be achieved by employing conductive carbon-based materials, combined with light modeling to optimize the electrode architecture.

Diffusional mediators can elicit the largest photocurrent enhancements of any strategy but only over short time scales. To extend their efficacy, in-depth mechanistic studies are needed to uncover their mode of operation, biological targets, interaction partners, and inactivation pathways.

For polymeric mediators, a deeper understanding of the intrinsic parameters that enable effective mediation are needed so that tailored structures can be designed rather than relying on polymers originally developed for other applications. Comparative studies of redox-active and conductive polymers are also essential to direct these future design strategies. Longevity studies on the operational stability of polymeric mediators and the physiological implications of embedding cells in a conductive matrix are crucial for evaluating the technological potential of this strategy.

An emerging strategy is the internalization of light-harvesting nanomaterials, such as gold nanoparticles, carbon dots, or quantum dots, to function as photosensitizers.^195^ These nanomaterials broaden solar spectrum absorption and contribute additional electrons to the cellular electron pool for EET. Liu et al. reported that *in situ* biosynthesized gold nanoparticles in cyanobacteria enhanced EET via photosensitization and additionally by acting as internal electron bridges, aiding electron export across cell membranes.^196^ However, the evidence presented suggests that the majority of gold nanoparticles are localized on the cell surface. This indicates that the photocurrent enhancement may arise primarily from improved biofilm conductivity and more efficient electron transfer at the cell–electrode interface rather than from photosensitization. To overcome this challenge, Kuruvinashetti et al. utilized a cell wall-deficient strain of the microalga *Chlamydomonas reinhardtii* to ensure gold nanoparticle internalization.^197^ The cytoplasm-localized gold nanoparticles enhanced light absorption and generated photoexcited electrons, improving device performance by 15.4%, highlighting that the efficacy of this approach depends on controlled nanoparticle distribution.

### 6.4 High Throughput Testing

The successful implementation of this approach necessitates exploring a large search space to find the optimal combination of cyanobacterial species and mutants, electrode materials and geometries, and chemical structures of mediators. As the library size of each component increases, the number of possible combinations grows exponentially, necessitating high throughput testing to effectively probe the search space. Such high throughput methodologies have revolutionized other fields such as drug discovery and more recently, organic synthesis and functional materials research. A tailored high-throughput screening platform for living photoanodes could similarly accelerate the discovery of synergistic interactions between polymer structures, electrode designs, cyanobacterial mutants and experimental conditions (pH, electrolyte, light source, and intensity) that deliver the highest and most stable photocurrent outputs. To enable this, bespoke electrochemical arrays with robust, reproducible electrochemical outputs and uniform illumination across each electrode need to be developed as currently available platforms lack sufficient customizability and illumination capacity. Cyanobacteria are conveniently well-suited for high throughput testing because their autotrophic nature enables straightforward and rapid cultivation for use in planktonic systems. Moreover, biofilm formation on electrodes can be achieved using simple dropcasting protocols in contrast to heterotrophic bacteria which often require time-consuming electrochemical activation. Cyanobacteria also exhibit reproducible, rapid and clear photocurrent responses making them straightforward and quick to assay. The screening process could be further accelerated, standardized and scaled up by automation with robotics which would also help reduce human error.

### 6.5 Potential of Living Photoanodes

Although still in the early stages of development, living photoanodes offer a promising strategy for sustainably harnessing and converting solar energy into electrons to perform useful work. Addressing the key challenges and following the roadmap outlined in this review could lead to breakthroughs needed to achieve long-term enhanced photocurrent outputs, approaching maximum theoretical values. In parallel, the analytical study of living photoanodes can provide new insights into fundamental biological processes of photosynthetic microorganisms or their components.^64,198^ Such revelations can guide optimization strategies for maximizing performance or inspire novel applications and research avenues. Similar approaches to those described here can be adapted for the development of living photocathodes which employ photosynthetic microorganisms as light-driven catalysts for CO_2_ fixation into value-added products. Realizing these advancements would bring within reach the possibility of incorporating these living anodes/cathodes into stand-alone solar-driven devices, unlocking a wide range of opportunities for the solar-powered synthesis of valuable products ranging from fuels to food and medicines, using self-sustaining biological systems augmented with rational, artificial interventions.

## Abbreviations

2D: two-dimensional
3D: three-dimensional
A: acceptor
ACNQ: 2-amino-3-carboxy-1,4-naphthoquinone
ARTO: alternative respiratory oxidase
ATP: adenosine triphosphate
BP-ITO: branched micropillar indium tin oxide
BQ: benzoquinone
CBB: Calvin–Benson–Bassham
Chl a: chlorophyll a
CN: carbon nitride
COX: cytochrome c oxidase
CPE: conjugated poly­(electrolyte)
Cyd: cytochrome bd-quinol oxidase
Cyt: cytochrome
Cyt *b*_6_ *f*: cytochrome *b*_6_ *f*
DCBQ: 2,6-dichloro-1,4-benzoquinone
DPP: diketopyrrolopyrrole
EASA: electroactive surface area
EET: extracellular electron transfer
EPS: extracellular polymeric substances
EQE: external quantum efficiency
F: Fe
FAD: flavin adenine dinucleotide
FB: Fe–S cluster
Fd: ferredoxin
FE: faradaic efficiency
FeCN: ferricyanide
Flv: flavodiiron
FNR: ferredoxin-NADP+ reductase
GM: genetically modified
H: haem
HPLC: high-performance liquid chromatography
IO-ITO: inverse opal indium tin oxide
LED: light-emitting diode
MP: microparticle
NADPH: nicotinamide adenine dinucleotide phosphate
NP: nanoparticle
OEC: oxygen-evolving complex
Omc: outer membrane cytochrome
OPP: oxidative pentose phosphate
P: primary electron donor
PAR: photosynthetically active radiation
Pc: plastocyanin
PDA: polydopamine
PEDOT: poly­(3,4-ethylenedioxythiophene)
PEDOT PSS: poly­(3,4-ethylenedioxythiophene) polystyrene sulfonate
PEDOT SDS: poly­(3,4-ethylenedioxythiophene) sodium dodecyl sulfate
PETC: photosynthetic electron transport chain
Pheo: pheophytin
POs: osmium-based redox polymer
PQ: plastoquinone
PSI: photosystem I
PSII: photosystem II
Q: quinone
QA: plastoquinone A
QB: plastoquinone B
RETC: respiratory electron transport chain
ROS: reactive oxygen species
SHE: standard hydrogen electrode
S-layer: surface layer
*Synechocystis*: *Synechocystis* sp. PCC 6803
TOF: turnover frequency
TON: turnover number
Tyr: tyrosine
η: overpotential
λ: wavelength
